# Sliding osteotomy for reconstruction of short post-gunshot mandibular defects < 2 cm: a prospective case series and narrative literature review

**DOI:** 10.1186/s12903-026-08713-9

**Published:** 2026-05-26

**Authors:** Bekir Osmanov, Danylo Kalashnikov, Sofiia Kozatska, Yurii Chepurnyi

**Affiliations:** Department of Maxillofacial Surgery, Bogomolets National Medical University, 1 Zoologichna Street, Office 411, Kyiv, 04119 Ukraine

**Keywords:** Mandibular reconstruction, Sliding osteotomy, Gunshot injury, Bone defect, Bone graft, Prosthetic rehabilitation

## Abstract

**Background:**

Reconstruction of short mandibular defects (< 2 cm) remains controversial, as these defects lie within a clinical “gray zone” between spontaneous healing and the need for surgical intervention. In cases of compromised soft tissues, particularly following gunshot injuries, conventional approaches such as bone grafting or microsurgical reconstruction may be suboptimal. This study aimed to evaluate the outcomes of sliding osteotomy (SO) techniques for reconstruction of post-gunshot short mandibular defects.

**Methods:**

This prospective case series included 10 patients with post-traumatic gunshot mandibular defects < 2 cm treated with SO techniques between March 2023 and September 2025. Clinical data, surgical characteristics, complications, and prosthetic outcomes were analyzed descriptively. A narrative literature review on SO techniques in mandibular reconstruction was also performed.

**Results:**

The mean defect length was 1.29 ± 0.22 cm. Most patients had a history of previous surgical interventions, including prior fracture fixation, failed bone grafting, and debridement. Horizontal SO was performed in 60.0% of cases and sagittal SO in 40.0%. Reconstruction failure due to non-union/fibrous union occurred in 2/10 patients (20.0%). Both patients required secondary mandibular reconstruction with a fibula free flap, which had been completed in one case at the time of evaluation.

**Conclusions:**

SO appears to be a technically straightforward option for reconstruction of short mandibular defects, particularly in patients with compromised soft tissue conditions or failed prior grafting. It may represent a salvage alternative within the reconstructive armamentarium for managing small but clinically critical defects.

**Trial registration:**

Not applicable. This was an observational prospective case series and did not involve prospective assignment of participants to an intervention.

## Background

The reconstruction of segmental mandibular defects remains a relevant clinical challenge despite significant advances in surgical techniques. Such defects arise following tumor resection, debridement procedures for necrosis and osteomyelitis, as well as high-energy trauma, including gunshot injuries, and are associated with disruption of mandibular continuity and significant functional impairment. Currently, reconstruction is primarily based on several key approaches, including the use of free bone grafts, particularly vascularized transplants, and, less commonly, distraction osteogenesis [1, 2].

One of the key factors influencing the choice of reconstructive technique is defect length. For mandibular defects exceeding 4–6 cm, most authors favor the use of vascularized flaps, most commonly the fibula free flap (FFF), whereas smaller defects are generally managed with non-vascularized bone grafts [3]. However, for smaller defects, particularly in the range of 0.5–2 cm, there is no consensus regarding the optimal treatment approach. Some authors do not consider such defects to be critical, suggesting that they may heal spontaneously. Although the definition of a “critical-sized defect” in the mandible remains debatable, it is evident that healing may occur under favorable clinical conditions. Nevertheless, certain types of defects, such as high-energy injuries resulting from combat trauma, as well as post-debridement defects, typically require surgical intervention [4, 5].

Small defects (< 2 cm) associated with significant periosteal and soft tissue damage, or with persistent chronic infection following a pathological mandibular fracture, are challenging to reconstruct using non-vascularized bone grafts. At the same time, microsurgical reconstruction may represent overtreatment or pose a risk of compromised vascular supply to a small bone segment [6]. The use of distraction osteogenesis requires complex devices and prolonged treatment under close clinical supervision, which significantly increases costs. In the setting of managing patients with combat-related injuries, this approach may be logistically impractical. Consequently, small defects (< 2 cm) fall into a “gray zone” of routine surgical decision-making and warrant further investigation.

In such complex clinical scenarios, alternative techniques capable of reconstructing ~ 1–2 cm bone defects may be effective, offering the advantages of technical simplicity and high predictability. The aim of this study was to evaluate the clinical outcomes of reconstructing post-traumatic gunshot mandibular defects using sliding osteotomy (SO) techniques, as well as to summarize the available evidence from the literature.

## Methods

This prospective case series included 10 consecutively enrolled patients with post-traumatic gunshot mandibular defects < 2 cm in length treated with sliding osteotomy techniques at the Head and Neck Pathology Center of Kyiv Regional Clinical Hospital (Kyiv, Ukraine) between March 2023 and September 2025. All patients who met the inclusion criteria during this period were included, and no eligible patients were excluded from the study. The study was conducted in accordance with the ethical principles of the Declaration of Helsinki (1964) and its subsequent amendments. The study protocol was reviewed and approved by the Bioethics Committee of Bogomolets National Medical University (Protocol No. 163).

The study included patients with post-traumatic gunshot mandibular defects < 2 cm in length who underwent reconstruction using SO techniques. The minimum postoperative follow-up period was 6 months.

Prospectively collected clinical data were obtained from the study database and medical records included patient age and sex, defect length and location, previous surgical interventions, type of osteotomy, operative time, fixation method, postoperative complications, and prosthetic rehabilitation. Successful reconstruction was defined as restoration of mandibular continuity with radiologically confirmed bone union 6 months postoperatively.

All procedures were performed under general anesthesia using standard extraoral approaches. All patients received perioperative antibiotic prophylaxis with amoxicillin; in cases with available microbiological testing, antibiotic therapy was adjusted according to culture and susceptibility results. Postoperative oral care included rinsing with a 0.05% aqueous chlorhexidine solution. Multislice computed tomography (MSCT) was performed within the first postoperative week and at 6 months after surgery.

The collected data were analyzed descriptively using absolute and relative (percentage) frequencies. For quantitative variables, the mean and standard deviation (M ± SD) were calculated.

A narrative (non-systematic) literature review was performed by the authors to identify and summarize previously published reports on sliding osteotomies in mandibular reconstruction. Electronic databases (PubMed, Scopus, and Google Scholar) were searched using combinations of keywords and terms consistent with Medical Subject Headings (MeSH), including “mandibular reconstruction”, “mandibular defect”, and “sliding osteotomy”, as well as related terms such as “intramandibular graft” and “sliding bone graft”. Additionally, references of selected articles were screened to identify further relevant studies not captured by the initial search. Only English-language articles describing clinical cases, case series, or original surgical techniques were considered. The selection of studies was based on their relevance to the topic and their contribution to understanding the indications, techniques, and outcomes of sliding osteotomies.

## Results

The series included 10 male patients with gunshot-related mandibular defects. The mean age was 41.0 ± 6.1 years (range, 33–55 years). The mean defect length was 1.29 ± 0.22 cm (range, 1.10–1.65 cm). In 7/10 (70.0%) cases, the defect was located in the lateral segments, while in 3/10 (30.0%) cases it involved the anterior mandibular body (Table 1).

**Table 1:** List of the patients in the study with description, surgical characteristics and outcomes

#	Age, y	Defect length, cm	Mandibular site	Previous procedures	Osteotomy type	Concomitant debridement (Yes/No)	Operative time, min.	Fixation method	Complications	Prosthetic rehabilitation	Notes
1	33	1.65	Lateral part	ORIF	Horizontal	Yes	130	Screws	None	Fixed dental prosthesis	Pre-existing mucosal defect; wound healing under iodoform gauze packing
2	35	1.33	Lateral part	ORIF, bone grafting with ICBG (*failure*)	Horizontal	Yes	55	PSI	None	Implant-supported prosthesis	Compromised dental implant position
3	55	1.12	Anterior part	ORIF, bone grafting with ICBG (*partial resorption*)	Horizontal	No	140	Miniplate & screws	None	Implant-supported prosthesis	Xenograft additionally used to fill the defect
4	40	1.64	Lateral part	None	Horizontal	Yes	120	Reconstructive plate & screws	None	Unrestored	A free-lying bone fragment was fixed in the donor site
5	41	1.20	Anterior part	ORIF, bone grafting with ICBG (*failure*)	Horizontal	Yes	80	Screws	Infection, non-union (fibrous union)	-	Reconstruction failure; patient scheduled for mandibular reconstruction with FFF
6	42	1.20	Anterior part	ORIF, bone grafting with ICBG and EORG (*failure*)	Sagittal	Yes	100	Reconstructive plate, miniplate & screws	None	Unrestored due to dental implant failure	None
7	43	1.48	Lateral part	ORIF	Sagittal	Yes	120	Reconstructive plate, miniplate & screws	None	Unrestored	None
8	38	1.12	Lateral part	ORIF, debridement	Sagittal	Yes	180	PSI	None	Unrestored	Trismus resolved
9	45	1.10	Lateral part	ORIF	Sagittal	No	145	Miniplate & screws	None	Not indicated	None
10	38	1.10	Lateral part	IMF, ORIF, debridement	Horizontal	Yes	130	Miniplate & screws	Non-union (fibrous union)	-	Reconstruction failure; successful mandibular reconstruction with an FFF at the subsequent stage.

Previous surgical interventions had been performed in 9/10 (90%) patients. Open reduction and internal fixation (ORIF) had been performed in 9/10 (90%) cases, and in 4/10 (40%) patients the defect had previously been reconstructed using an iliac crest bone graft (ICBG).

**Fig. 1:**
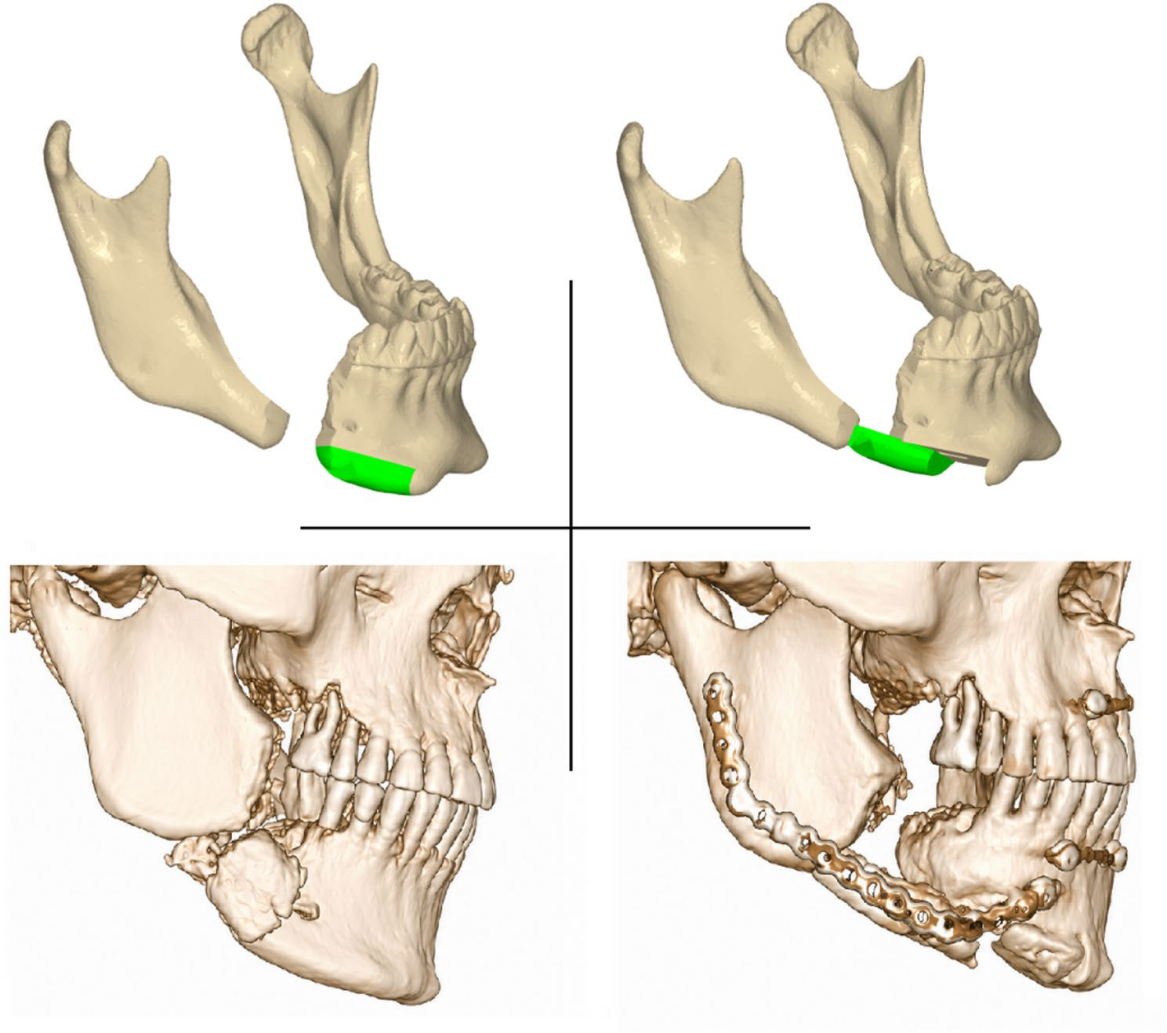
Horizontal sliding osteotomy: schematic illustration and 3D MSCT reconstruction before and after surgery (patient No. 4)

**Fig. 2:**
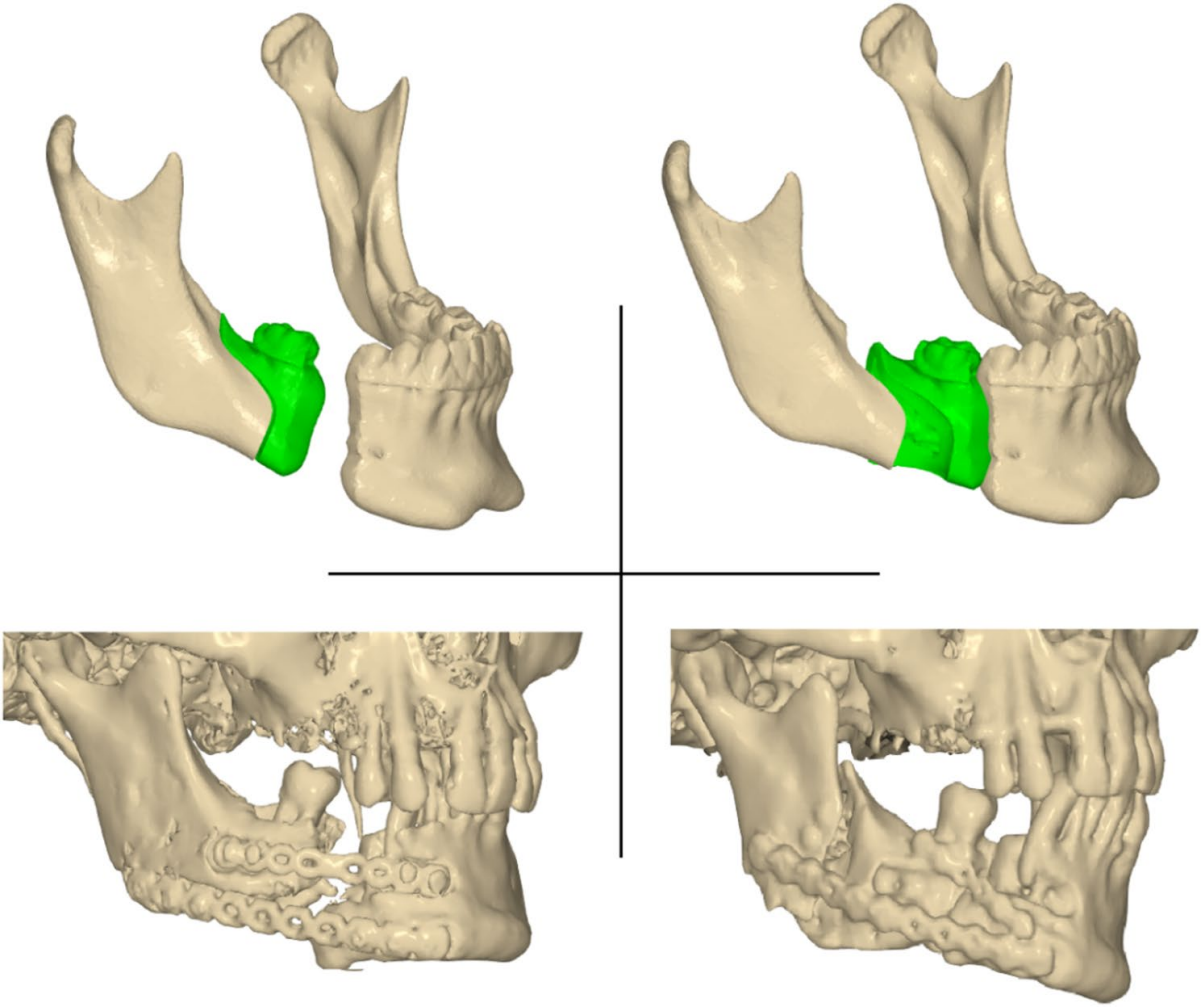
Sagittal split (sliding) osteotomy technique for reconstruction of a short mandibular defect: schematic illustration and 3D MSCT reconstruction before and after surgery (patient No. 7)

The predominant SO technique was horizontal, performed in 6/10 (60.0%) cases (Fig. 1), whereas a sagittal split approach was used in 4/10 (40.0%) (Fig. 2). Concomitant debridement was carried out in 8/10 (80.0%) cases, involving removal of non-viable bone fragments (sequestra) or disintegrated bone grafts. The mean operative time was 120 ± 35 min (range, 55–180 min). The variability in operative time reflected differences in the extent of surgery and concomitant procedures. In some cases, SO was combined with debridement and removal of previous fixation hardware. In patient No. 8, coronoid process resection was additionally performed during the same procedure to treat trismus, contributing to the longest operative time.

For fixation, plates (reconstruction plates or miniplates) with screws were most commonly used in 6/10 (60.0%) cases, followed by screws alone in 2/10 (20.0%) and patient-specific implants (PSI) in 2/10 (20.0%).

Postoperative complications were observed in 2/10 patients (20%). Bone union at 6 months was radiologically confirmed in 8 of 10 patients; patients No. 5 and No. 10 developed non-union/fibrous union and were classified as reconstruction failures. One patient developed an infection followed by non-union, resulting in reconstruction failure; therefore, the patient was scheduled for mandibular reconstruction with a FFF. Another patient developed isolated non-union and underwent FFF reconstruction at a subsequent stage. Prosthetic rehabilitation was completed in 3 of the 8 successful reconstructions, including implant-supported restorations in 2 patients and a fixed dental prosthesis in 1 patient. Among the remaining five successfully reconstructed patients, prosthetic rehabilitation was not indicated in one case because of a distal defect in the second molar region without opposing dentition. In one case, prosthetic rehabilitation is ongoing after dental implant disintegration. In three other cases, rehabilitation was planned but had not yet been performed at the time of evaluation because the patients continued military service and had limited availability for further staged treatment. The follow-up period for prosthetic outcomes extended up to 2 years.

Although all patients had post-traumatic scarring and presumed periosteal compromise, specific documented soft-tissue events requiring separate reporting were present in two cases. In one patient, soft tissue volume had been previously restored during reconstruction of the bone defect with an ICBG using a free anterolateral thigh flap (ALTF). In another case, a mucosal defect was present both before and after the SO and healed by secondary intention.

## Discussion

The reconstruction of short (~ 1–2 cm) mandibular defects requires consideration not only of defect length, but also of the biological conditions required for spontaneous healing. Experimental studies have shown that the threshold for a critical-sized mandibular defect varies substantially depending on periosteal preservation and soft tissue vascularity. In canine and minipig models, preservation of the periosteum allowed regeneration of considerably larger defects, whereas removal of periosteal coverage made even 15–20 mm defects critical [7, 8]. Comparable findings have also been reported in rabbit models [9]. Thus, there is no universal size threshold for a critical mandibular defect; rather, healing potential is largely determined by the condition of the surrounding vascularized tissues.

This concept is also supported by clinical studies of mandibular reconstruction using bone grafts, where the success of reconstruction is strongly influenced by the proximity and quality of surrounding soft tissues as a source of vascular ingrowth [6, 10]. Accordingly, in our series, despite the relatively small mean defect length of 1.29 ± 0.22 cm, the post-gunshot soft tissue scarring, presumed periosteal compromise, and impaired vascularity reduced the potential for spontaneous bone regeneration.

Undoubtedly, in the presence of adequate soft tissue coverage in the region of an existing or potential defect, the SO modifications used in this series should not be considered first-line treatment options. As noted by Hell BH (2017) [11], who reported reconstruction of the anterior mandibular body using bilateral sagittal osteotomy with advancement of the lingual cortical plates following oncologic resection, such approaches should be considered “emergency procedures” reserved for selected clinical cases in which standard methods are limited. However, in the presence of compromised soft tissues, as observed in post-radiation or post-gunshot defects, their use may become considerably more justified.

In our series, the use of local reconstructive techniques was primarily driven by the following clinical scenarios and factors:

1. The length of the mandibular bone defect did not exceed 2 cm;
2. Reconstructions were performed in patients with post-traumatic gunshot defects characterized by periosteal loss with significant scarring, impaired soft tissue vascularization in the injured area, and frequent presence of infection [12, 13];
3. At the primary stage of treatment, during ORIF, mandibular ramus fragments were often fixed in a displaced position with occlusal disturbance; upon repositioning into the correct alignment, a true defect became apparent;
4. First-line methods for reconstruction of short defects, particularly the use of ICBG, had already proven unsuccessful due to significant graft resorption or loss resulting from infectious complications.

One of the earliest detailed descriptions of this technique, known as the *Intramandibular Sliding Myoosseous Graft*, was provided by Browne and McQuarrie (1985) [14]. They proposed a method of opposing mobilization of bone segments from the inferior border of the lateral mandibular body and ramus while preserving the muscular attachments of the medial pterygoid and masseter muscles. Prior to the era of microvascular bone transplantation, this approach could be considered one of the primary reconstructive options. The authors reported that this technique could be used to reconstruct mandibular defects up to 8 cm in length. However, according to Pereira et al. (1997), the practical limit for its application is defects not exceeding 7 cm [15].

In many classical publications, these techniques have been described for complete reconstruction of entire mandibular segments, particularly the anterior region, to address extensive defects measuring up to 6–8 cm [11, 15–17]. In contrast, in more recent studies, including the present series, this approach is considered appropriate primarily for defects not exceeding 4 cm.

Subsequently, Salins and Rao (1997) [16] described a modified sliding osteotomy technique involving splitting of the mandibular ramus segment, achieving reconstruction of an 8 cm anterior defect via an intraoral approach and proposing this method as an optimal surgical solution in such clinical scenarios.

The study by Verdaguer et al. (2001) [17] summarized and expanded previously described SO techniques, presenting outcomes in 14 clinical cases. The series included five osteotomy variants for reconstruction of lateral and anterior mandibular defects: C-shaped, horizontal (single, double, bilateral sliding), and sagittal sliding, the latter being well known from orthognathic surgery as sagittal split osteotomy. Overall, the authors concluded that SO techniques are best suited for short defects (< 4 cm) or in the presence of general contraindications to more complex reconstructive procedures.

Despite the variability of mandibular osteotomy techniques, all are based on a fundamental principle - preservation of the periosteum and muscular attachments to the osteotomized segments (medial pterygoid, masseter, mylohyoid, digastric, and geniohyoid muscles), which is critical for vascularization and viability of the bone segments. In our experience, the extent of the defect amenable to reconstruction was primarily determined by the ability to mobilize the osteotomized segment without disrupting these muscular attachments.

In the present series, the choice between horizontal and sagittal sliding osteotomy was mainly determined by defect configuration and the possibility of preserving vascularized periosteal and muscular attachments to the osteotomized segment. Horizontal sliding osteotomy was preferred when a pedicled mandibular fragment could be mobilized with preserved muscular attachments, particularly the mylohyoid muscle in appropriate regions. Sagittal sliding osteotomy was selected when there was uncertainty regarding preservation of these attachments to the potential horizontal fragment, or when sagittal advancement provided more favorable bone-to-bone contact and less vertical height discrepancy.

Overall, the published reports summarized in Table 2 generally demonstrated favorable outcomes regarding survival and union of the mobilized bone segments. Despite a relatively low complication rate, cases of bone resorption and fracture, osteomyelitis with orocutaneous fistula formation, fibrous union, as well as hardware failure or instability have been reported [14, 17, 18]. In our series, no resorption of the osteotomized and mobilized segments was observed. However, two cases of unilateral non-union/fibrous union were identified in patients No. 5 and No. 10. Both failures occurred after horizontal sliding osteotomy, but the cases were otherwise heterogeneous: defect lengths were 1.20 cm and 1.10 cm, respectively, and were not the largest in the series; one defect was anterior and the other lateral; infection was present in one case only; and fixation methods differed. All procedures were performed by the same surgeon, reducing operator variability. We hypothesize that insufficient bone-to-bone contact between the mobilized and recipient fragments may have contributed to non-union. However, the small sample size precludes identification of definite risk factors.

**Table 2:** Published clinical reports and case series on sliding osteotomy techniques in mandibular reconstruction

Author(s)	Year	Case number	Sliding osteotomy type / method (by author)	Defect length	Mandibular body site	Etiology	Complications	Prosthetic rehabilitation
Browne & McQuarrie [14]	1985	9	Horizontal sliding osteotomy	Up to 8 cm	Anterior part	Oncology (carcinoma)	Orocutaneous fistula; fibrous union (3 cases); local osteoradionecrosis	Not reported
Pereira et al. [15]	1997	3	Horizontal sliding osteotomy	Up to 7 cm	Anterior part with involvement of lateral parts	Oncology (squamous cell carcinoma)	Bone fragment fracture (1 case)	Not reported
Salins & Rao [16]	1997	1	Horizontal sliding ramus split osteotomy	8 cm	Anterior part with involvement of lateral parts	Odontogenic keratocyst	Reconstructive plate fracturing	The placement of dental implants was being considered
Verdaguer et al. [17]	2001	14	5 techniques of sliding osteotomy: C-shaped, horizontal (single/double/bilateral) and sagittal	Mean 3.8 cm (1.5–7 cm)	Lateral and anterior parts	Oncology (squamous cell carcinoma) − 13 cases, 1 case of gunshot injury	Osteomyelitis, osteoradionecrosis (2 cases); bone fragment fracture (1 case); fixation screws failure (1 case); non-union (fibrous union)	Removable dentures in 5 patients, implant-supported dentures in 3 patients
Bianchi et al. [19]	2009	1	Horizontal sliding osteotomies + OCRFFF	Not reported	Anterior part with involvement of lateral parts (area between 1st molars)	Oncology (squamous cell carcinoma)	No complications were observed	Not reported
Chen et al. [18]	2011	13	Horizontal sliding osteotomies + ALTF (9) / RFFF (3) / tongue flap (1)	Mean 4.6 cm (3–6 cm)	Lateral part	Oncology (squamous cell carcinoma)	Two cases of bone fragments non-vitality: resorption of bone fragment (with subsequent malunion, titanium plate and screws loosening) and osteomyelitis with orocutaneous fistula; wound infection; trismus;	Implant-supported removable dentures in 1 patient
Hell B.H. [11]	2017	1	Sagittal split osteotomies with advancement of lingual cortical plates + additional osteotomies of the fragments	Not reported	Anterior part with involvement of lateral parts	Oncology (squamous cell carcinoma)	Dehiscence, fistula	Dental implantation (6 impl.) performed
Tay HW et al. [20]	2021	1	Horizontal sliding osteotomy + sagittal sliding osteotomy + EORG + RFFF	7 cm	Anterior and lateral mandible parts	Oncology (squamous cell carcinoma)	Resorption of the free bone graft	Not reported
Grab et al. [21]	2023	1	Sagittal split osteotomy + augmentation with cancellous bone from tibia	~ 2 cm	Lateral part	Multifragment mandibular fracture	No complications were observed	Not indicated
Liu HP et al. [22]	2024	1	Sagittal split osteotomy + RFFF	4 cm	Lateral part	Oncology (squamous cell carcinoma)	Bone fragments and plate fracturing 2 years postoperatively	Not reported

Although no complications were observed in patients treated with sagittal sliding osteotomy, this finding should be interpreted with caution. The present study is a case series and was not designed to compare different osteotomy techniques. A larger cohort is required to determine whether one approach has any clinical advantage over the other using appropriate statistical analysis.

Secondary management of comminuted gunshot fractures was the most common indication for SO in our series. In these injuries, extensive fragmentation, tissue contamination, and prior internal fixation may compromise blood supply and predispose to osteomyelitis. After debridement, SO allowed immediate reconstruction of the resulting short defect using a pedicled bone segment. Although external fixation may be useful in comminuted fractures with infection or soft tissue deficiency, its use in high-intensity combat settings may be limited by device availability and the need for close postoperative monitoring [23, 24].

It should be noted that among the numerous published case series and reports, only Verdaguer et al. (2001) [17] described a single patient with a post-traumatic gunshot mandibular defect successfully reconstructed using a SO technique. Therefore, our series represents a unique application of this method in a cohort of patients with gunshot defects.

The second most common indication for SO in our series was failure of iliac crest autografts due to infectious complications or significant resorption, in some cases exceeding 60–70% [25, 26], resulting in failure to restore mandibular continuity. The rationale for considering SO as a salvage option was partly based on our previous experience with non-vascularized iliac crest grafting in combat-related mandibular defects [6]. In that cohort, graft reconstruction was associated with a high complication rate: the overall success rate was 59.3%, decreasing to 14.3% in cases with minor soft-tissue defects, while sufficient soft-tissue coverage increased the success rate to 75.0%. However, that study did not specifically analyze defects < 2 cm, and the smallest defect was 2 cm. Therefore, a direct comparison between SO and ICBG for defects < 2 cm cannot be made. In the present series, 4 of 10 patients had already experienced failed ICBG reconstruction. Consequently, SO was selected as an alternative reconstructive pathway rather than repeating bone grafting in a compromised recipient site. In such cases, SO allowed restoration of mandibular continuity during revision surgery without repeated graft harvesting or transfer of a free graft into a compromised soft tissue environment. Another important advantage of this technique is the reduced dependence of the viability of the pedicled bone segment on the condition and integrity of the oral mucosa, which is a critical factor in reconstruction using ICBG [27–29]. Based on our experience, ICBG remains preferable for short mandibular defects when soft-tissue coverage is adequate, infection is controlled, and reconstruction can be performed in a delayed setting. SO may be considered in short defects after failed ICBG, when debridement or necrectomy is required, or when local soft-tissue conditions are compromised. FFF was reserved for secondary reconstruction after failure of graft-based or local reconstruction, or when further resection resulted in a larger defect, rather than as a first-line option for defects < 2 cm.

In one case (patient No. 3), the voids between the integrated bone graft and the osteotomized mandibular segment were filled with a xenogeneic bone substitute (1–2 mm granules) to improve conditions for subsequent dental implantation. A similar approach was reported by Grab et al. (2023), who used a sagittal SO during secondary treatment of a mandibular fracture, with cancellous bone harvested from the tibia for additional augmentation of the split bone segments [21].

Several authors have also incorporated SO into combined reconstructive approaches with vascularized soft tissue flaps, including RFFF, ALTF, tongue flaps, and OCRFFF [18–20, 22]. These reports support the use of SO as a compromise or adjunctive option when conventional osseous free flap reconstruction is limited. In our series, ALTF was used in patient No. 3 at a prior stage for soft tissue reconstruction.

Regarding the limitations of SO technique, most authors report that even when used for reconstruction of larger defects, the best aesthetic and functional outcomes are achieved in defects < 4 cm. Restoration of a normal facial profile in larger defects, particularly in the anterior region, is considered nearly impossible, as it is associated with reduced vertical height, profile flattening, development of a square chin, and visible mandibular asymmetry [11, 14, 16, 18].

In our series, no facial contour deformities were observed, likely due to the small size of the bone defects and osteotomized segments. In contrast, contour irregularities in our patients were primarily related to soft tissue scarring resulting from the initial gunshot injuries. Notably, bone regeneration and remodeling were observed at the donor site, rendering the osteotomy site of the bone segment nearly indistinguishable on postoperative follow-up MSCT scans.

Horizontal sliding osteotomy may result in reduced vertical height of the reconstructed mandibular segment. In our series, the primary goal was restoration of mandibular continuity and bone union rather than complete restoration of alveolar height. When relevant for prosthetic rehabilitation, this discrepancy was managed individually: by using a fixed dental prosthesis supported by teeth adjacent to the defect, by placing dental implants on both sides of the reconstructed area, or by planning secondary bone augmentation when additional vertical bone volume was required for implant-supported rehabilitation.

Non-union/fibrous union has also been reported in similar studies, including those by Browne & McQuarrie (1985) [14], Verdaguer et al. (2001) [17]. Potential contributing factors include the stress-shielding effect associated with rigid fixation using reconstruction plates [30–32], as well as the limited bone-to-bone contact area between fragments, as discussed above [33, 34]. Nevertheless, this issue is complex and warrants further investigation.

A mucosal defect in patient No. 1 should be noted separately. It was not considered a postoperative complication, as the defect was present prior to surgery as a consequence of previous failed ORIF complicated by infection and osteomyelitis. Debridement was therefore performed during the same surgical procedure, and the defect healed by secondary intention under iodoform packing. Hell et al. (2017) [11] described the urgent use of a nasolabial flap to cover exposed bone in cases of mucosal dehiscence in the early postoperative period. In contrast, our approach was based on the premise that bone segments used in SO are less dependent on the integrity of the oral mucosa compared to free bone grafts [27–29].

This study has several limitations. First, although it represents one of the larger series reported in the literature, the small sample size allowed only descriptive analysis without statistical evaluation of outcomes or potential risk factors for non-union. Second, while the homogeneity of this case series, comprising exclusively patients with traumatic gunshot defects, can be considered a strength, it also limits the generalizability of the findings to other etiologies. Third, the study lacks a control group or direct comparison with alternative treatment options. Fourth, potential selection bias should be acknowledged, as the decision to perform SO rather than another reconstructive technique was based on surgeon judgment and individual clinical conditions. Finally, radiological bone union at 6 months represents a relatively short endpoint, and further studies are required to assess long-term stability, late complications, and completion of prosthetic rehabilitation.

## Conclusion

In selected cases of post-gunshot mandibular injuries, even short segmental defects may be clinically critical and require surgical reconstruction. Sliding osteotomy appears to be a feasible and technically straightforward salvage option for reconstruction of short defects, particularly after failed bone grafting or in compromised recipient sites affected by scarring or infection. Its potential advantages include technical simplicity, the possibility of simultaneous debridement, and avoidance of an additional donor site. Further comparative studies with longer follow-up are required to define its indications and long-term stability.

## Data Availability

Data supporting the findings of this study are available from the corresponding author (BO) upon reasonable request.

## Abbreviations

ALTF: Anterolateral thigh flap
EORG: External oblique ridge
FFF: Fibula free flap
ICBG: Iliac crest bone graft
IMF: Intermaxillary fixation
MSCT: Multislice computed tomography
SO: Sliding osteotomy
ORIF: Open reduction and internal fixation
OCRFFF: Osteocutaneous radial forearm free flap
PSI: Patient–specific implant(s)
RFFF: Radial forearm free flap
